# The LXR-623-induced long non-coding RNA LINC01125 suppresses the proliferation of breast cancer cells via PTEN/AKT/p53 signaling pathway

**DOI:** 10.1038/s41419-019-1440-5

**Published:** 2019-03-13

**Authors:** Weijun Wan, Yongying Hou, Ke Wang, Yue Cheng, Xia Pu, Xiufeng Ye

**Affiliations:** Department of Pathology, Chongqing Medical University, Chongqing, 400016 China

## Abstract

LXR-623 (WAY-252623), a liver X receptor agonist, reduces atherosclerotic plaque progression and remarkably inhibits the proliferation of glioblastoma cells, owing to its brain-penetrant ability. However, the role of LXR-623 against the proliferation of other cancer cells and the underlying mechanism remain unknown. Long non-coding RNAs (lncRNAs) serve as novel and crucial regulators that participate in cancer tumorigenesis and diverse biological processes. Here, we report a previously uncharacterized mechanism underlying lncRNA-mediated exocytosis of LXR-623 via the phosphatase and tensin homolog (PTEN)/protein kinase B (AKT)/p53 axis to suppress the proliferation of cancer cells in vitro. We found that LXR-623 significantly inhibited the proliferation and induced apoptosis and cell cycle arrest at S phase in breast cancer cells in a concentration- and time-dependent manner. Experiments using a xenograft mouse model revealed the inhibitory effects of LXR-623 on tumor growth. We used lncRNA microarray to investigate the potential genes regulated by LXR-623. As a result, LINC01125 was found to be significantly upregulated in the cells treated with LXR-623. Gain- and loss-of-function assays were conducted to investigate the anti-proliferation role of LINC01125. LINC01125 knockdown resulted in the inhibition of the cytotoxic effect of LXR-623; in contrast, LINC01125 overexpression significantly enhanced the effect of LXR-623. LXR-623 and LINC01125-mediated anti-growth regulation is, at least in part, associated with the participation of the PTEN/AKT/mouse double minute 2 homolog (MDM2)/p53 pathway. In addition, SF1670, a specific PTEN inhibitor with prolonged intracellular retention, may strongly block the anti-proliferation effect induced by LXR-623 and LINC01125 overexpression. Chromatin immunoprecipitation (ChIP) assay results suggest that p53 binds to the promoter of LINC01125 to strengthen the expression of the PTEN/AKT pathway. Taken together, our findings suggest that LXR-623 possesses significant antitumor activity in breast cancer cells that is partly mediated through the upregulation in LINC01125 expression and enhancement in apoptosis via the PTEN/AKT/MDM2/p53 pathway.

## Introduction

Breast cancer (BC) is one of the most common cancers and accounts for about 30% of the cancer cases in females worldwide. It is ranked as the second most common cause of cancer-related deaths^1,2^. Treatment strategies for BC, including breast-conserving surgery or mastectomy, chemotherapy, radiation therapy, hormone therapy, and other new therapies, are based on individual characteristics of clinical pathology^3^. However, many patients with BC experience relapse within a few years, and the long-term mortality rate remains high. Therefore, new therapeutic approaches and discovery of patient-friendly therapeutics that are safe and efficacious are desirable^4,5^.

Liver X receptors (LXRs) are nuclear receptors that induce the expression of the transporters responsible for promoting cholesterol efflux, leading to the reduction in atherosclerosis. LXRs are significant regulators of the fatty acid and glucose homeostasis as well as the immune system^6,7^. Recent reports have revealed that blastic plasmacytoid dendritic neoplasm cell lines restored LXR target gene expression and increased cholesterol efflux via the upregulation in the expression of adenosine triphosphate-binding cassette (ABC) transporters, ABCA1 and ABCG1, in response to LXR agonist treatment^8^. In addition, LXR agonist may regulate the progression of prostate cancer through suppressor of cytokine signaling 3^9^ and reduce protein kinase B (Akt) phosphorylation in BC^10^. LXR-623, a novel LXR agonist and clinically effective anti-atherogenic agent, could significantly kill glioblastoma cells in an LXRβ- and cholesterol-dependent manner, cause tumor regression, and prolong the survival of mouse models, owing to its low toxicity and high brain-penetrant ability^11^. However, little is known about the antitumor impact of LXR-623 on other cancers.

Long non-coding RNAs (lncRNAs), a group of transcripts greater than 200 nucleotides in length, are involved in a variety of pathophysiological and biological processes in the human body, especially in the tumorigenesis and progression of cancer. Hence, lncRNAs have attracted the attention of researchers. Accumulating evidence indicates that the aberrant expression of lncRNAs is associated with tumorigenesis through multiple biological mechanisms involving epigenetic, transcriptional, and post-transcriptional alterations^12–14^. For instance, HOTAIR is a lncRNA that plays a key role in several cancers such as breast, gastric, colorectal, and cervical cancers and the expression level of HOTAIR is a potential biomarker for diagnostic and therapeutic purposes^15,16^.

Here, we hypothesize that lncRNAs may play a key role in the regulation of LXR-623-induced antitumor effects. In this study, we used BC cells and animal models to detect the antitumor activity of LXR-623 and investigated the underlying molecular mechanism. LXR-623 was shown to suppress the proliferation of BC cell lines and inhibit the growth of tumor xenografts. This action was associated with the expression of a lncRNA called LINC01125. Furthermore, the knockdown of LINC01125 blocked the inhibitory effects of LXR-623, whereas LINC01125 overexpression sensitized the BC cells to LXR-623. The results of the present study revealed that LINC01125 mediate the LXR-623-induced anti-proliferation effect by regulating phosphatase and tensin homolog (PTEN) and AKT/p53 pathways. Therefore, this study provides a new insight into the chemopreventive mechanism associated with the LXR agonist application for cancer treatment.

## Results

### LXR-623 suppresses the proliferation of BC cell lines

**Fig. 1:**
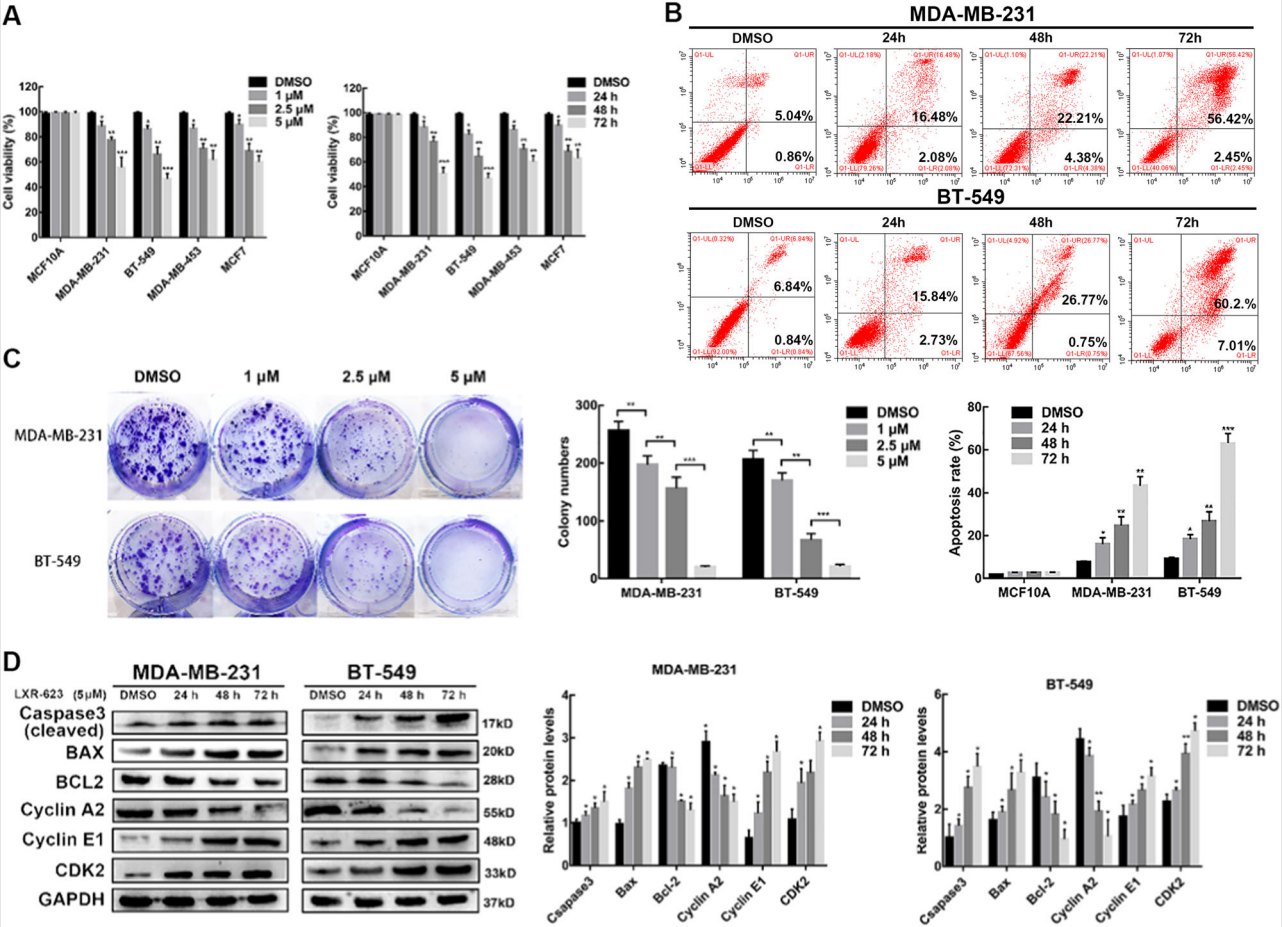
LXR-623 suppresses the proliferation in breast cell lines. **a** CCK8 assay was used to detect the inhibitory effect of LXR-623 in breast cancer cells for 72 h (left panel) and at 5 μM (right panel) compared to MCF10A cells. DMSO discs were used as negative controls. **b** Flow cytometry analysis showed LXR-623 (5 μM) increases cell apoptosis. **c** The ability of tumor cells to form colonies was assessed 14 days post-treatment with the indicated concentrations of LXR-623. **d** MDA-MB-231 and BT-549 cells were treated with LXR-623 (5 μM) for the indicated times, and whole cell extracts were prepared and analyzed using the indicated antibodies. Data shown are mean ± SD of three independent experiments. *P < 0.05, **P < 0.01, ***P < 0.001

To evaluate the effect of LXR-623 on BC cells and a normal epithelial breast cell line MCF-10A, we measured the cell viability using the cell counting kit 8 (CCK8) assay after the treatment of cells with LXR-623 at different concentrations (1, 2.5, and 5 μM) for 24, 48, and 72 h. As a result, we found that LXR-623 significantly suppressed the viability of MCF-7, MDA-MB-231, BT549, and MDA-MB-453 cells in a dose- and time-dependent manner (Fig. 1a). Furthermore, flow cytometry was used to detect the number of apoptotic cells after exposure to LXR-623 at 5 μM for 24, 48, and 72 h. In comparison with the cells treated with dimethyl sulfoxide (DMSO; control group), MDA-MB-231 and BT549 cells treated with LXR-623 for 72 h showed almost 60% increase in the rate of apoptosis (Fig. 1b). Then, colony formation assay revealed the significant inhibition in the colony-forming ability of MDA-MB-231 and BT549 cells after treatment with the tested drug at a concentration of 1, 2.5, and 5 μM (Fig. 1c). These data suggest that LXR-623 could significantly inhibit proliferation and induce apoptosis in BC cells. We tested the expression of apoptosis-related proteins by western blot analysis and found that the expression of activated (cleaved) caspase-3 and BAX was upregulated and that of B-cell lymphoma 2 (BCL2) was downregulated in a time-dependent manner (Fig. 1d). In addition, we investigated the effect of LXR-623 at 5 μM concentration for 24, 48, and 72 h on cell cycle progression by flow cytometry and observed cell cycle arrest at the S phase in MDA-MB-231 and BT549 cells as compared to the control group (Figure S1a). The number of cells in the G1 stage decreased while that in the S stage increased in LXR-623-treated group versus the control group, indicating that the cells become insensitive to LXR-623-induced quiescence during S phase. As LXR-623 may induce cell cycle arrest, we tested the protein levels of cyclin E1, cyclin-dependent kinase 2 (CDK2), and cyclin A2 with western blot analysis. As shown in Fig. 1c, the expressions of cyclin E1 and CDK2 were upregulated and cyclin A2 expression was downregulated, eventually manifesting the transition from the G1 to S phase followed by quiescence in the S phase in a time-dependent manner.

### Antitumor effects of LXR-623 in vivo

**Fig. 2:**
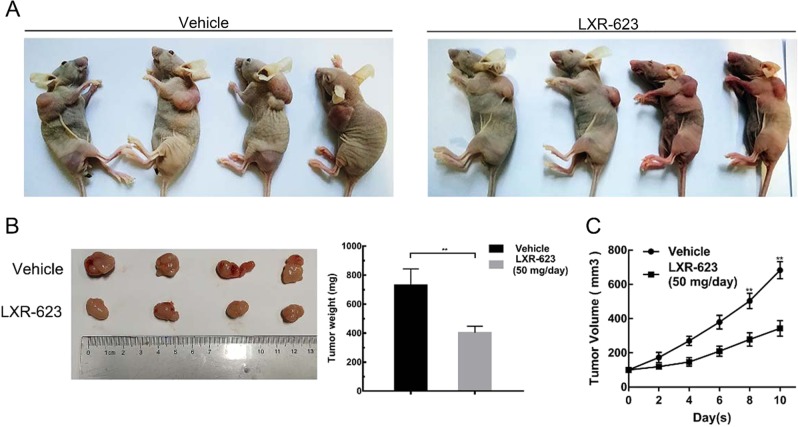
Antitumor effects of LXR-623 in vivo. 2 × 10^6^ MDA-MB-231 cells were individually injected to the dorsal region and mice were randomly divided into two groups when the tumors reached a size of approximately 100 mm^3^ in all mice. Mice were treated with vehicle or LXR-623 50 mg/kg PO daily. **a** The mice were sacrificed and photographed (*n* = 4). **b** Tumor weights were also shown. **c** Tumor volumes were assessed every other day during drug treatment (*V* = *AB*^2^/2). *P < 0.05, **P < 0.01

To validate the antitumor effect of LXR-623 in vivo, female nude mice were subcutaneously injected with MDA-MB-231 cells to induce tumor formation. The mice were randomly divided into two groups once the tumor volumes reached about 100 mm^3^. During the treatment period of 10 days, tumor weight and volume significantly decreased for mice treated with LXR-623 as compared to those from the control group (Fig. 2a, b). LXR-623 could markedly inhibit the tumor growth (Fig. 2c).

### LXR-623-induced LINC01125 expression is downregulated in human BC tissues and cells

**Fig. 3:**
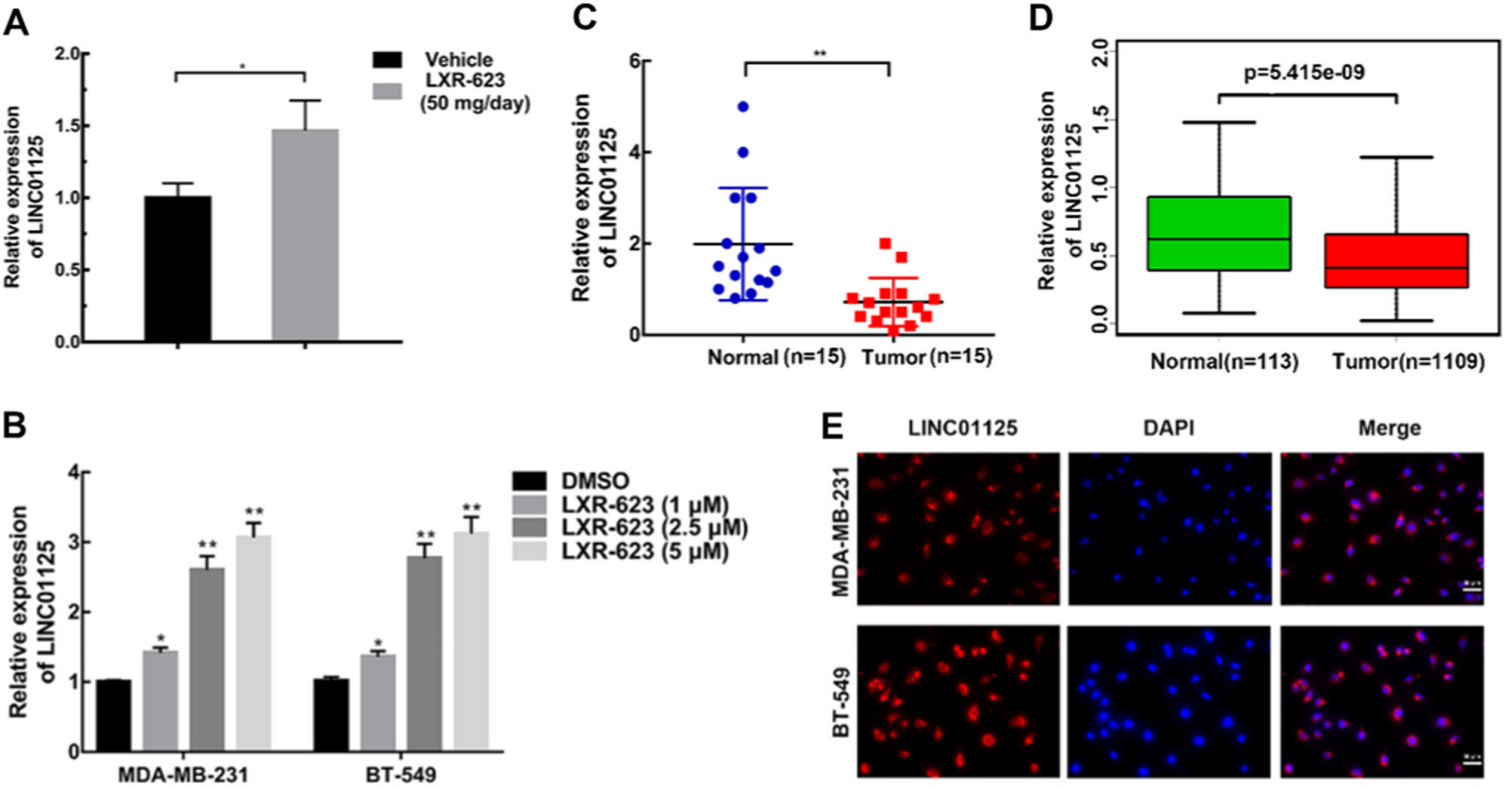
LXR-623-induced LINC01125 is downregulated in human breast cancer tissues and cells. LncRNA array detected the differentially expressed genes regulated by LXR-623 at 5 μM for 48 h. **a** LINC01125 expression levels were measured in xenograft tumors and **b** in MDA-MB-231 and BT-549 cells treated with LXR-623 for 48 h at the indicated concentrations by RT-qPCR. **c** The expression of LINC01125 in the 15 pairs of breast cancer tissues and adjacent non-cancerous tissues by RT-qPCR. **d** TCGA database analysis of LINC01125 in breast cancer. **e** Representative images of FISH of LINC01125 in MDA-MB-231 and BT-549 (scale bar, 50 μm). *P < 0.05, **P < 0.01

To identify the candidate lncRNAs involved in LXR-623-mediated tumor suppression, we used the lncRNA microarray to evaluate the differentially expressed genes regulated by LXR-623 at 5 μM concentration for 48 h in MDA-MB-231 cells. lncRNA microarray results revealed significant changes in the expressions of 943 human lncRNAs after treatment with LXR-623. Of these human lncRNAs, 433 were upregulated and 510 were downregulated. qRT-PCR results confirmed the increase in the expression of LINC01125 mediated by LXR-623 in the xenograft tumors as well as MDA-MB-231 and BT549 cells in a dose-dependent manner (Fig. 3a, b). LINC01125 expression level was downregulated in tumors as compared with the corresponding normal tissues (Fig. 3c). We used The Cancer Genome Atlas (TCGA) database to analyze the expression of LINC01125 (Fig. 3d) and found that LINC01125 expression was downregulated in BC tissues. qRT-PCR was used to confirm the downregulation of LINC01125 expression in MDA-MB-231, BT549, MCF-7, and MDA-MB-453 cells than in MCF-10A cells. Immunofluorescence assay consistently showed that LINC01125 was more enriched in the cytoplasm of MDA-MB-231 and BT549 cells (Fig. 3e). Therefore, LINC01125 may play an important role as a suppressor gene in BC.

### LINC01125 plays a critical role in LXR-623-induced apoptosis

**Fig. 4:**
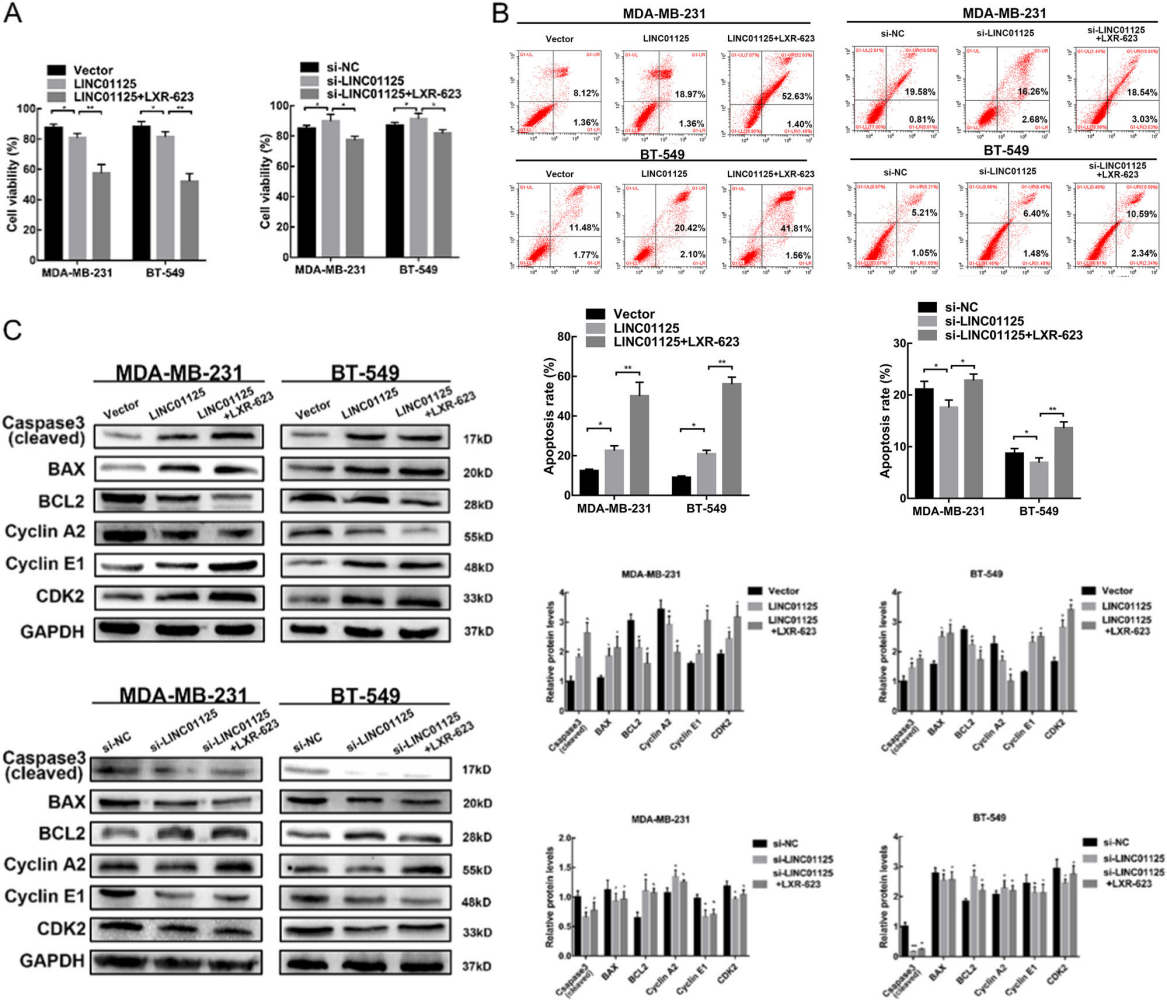
LINC01125 plays a critical role in the LXR-623-induced apoptosis. **a**, **b** Effect of LINC01125 and combined effect of LXR-623 (5 μM) and LINC01125 siRNAs or overexpression vectors on breast cancer cell proliferation measured by CCK8 assays and flow cytometry analysis. **c** Western blot showed the effect of LINC01125 overexpression and knockdown and combined with LXR-623 (5 μM) in apoptosis and cell cycle-related protein levels. All experiments were performed in triplicate, and data are expressed as the mean ± SD of at least three independent experiments. *P < 0.05, **P < 0.01

To assess the potential functional role of LINC01125 in LXR-623-induced tumor suppression, we examined the impact of LINC01125 overexpression and knockdown in BC cell lines. As shown in Fig. 4a, b, the knockdown of LINC01125 with a small interfering RNA (siRNA) led to an increase in the proliferation of tumors; however, the treatment of these transfected cells with LXR-623 (5 μM) partially blocked the inhibitory effects of LXR-623, as revealed by the CCK8 and apoptosis assays. In contrast, cell viability significantly decreased for those cells overexpressing LINC01125 as compared with the negative control group. Upon treating cells with LXR-623, the apoptosis rate was dramatically increased. Western blotting results showed that LXR-623 induced the expression level of cleaved caspase-3 and BAX and suppressed the expression of BCL2. This effect may be strengthened through the overexpression of LINC01125 or partly blocked through LINC01125 knockdown (Fig. 4c). Next, the knockdown of LINC01125 slightly reduced the number of cells in the S phase, but LINC01125 overexpression increased the number of cells in the S phase and strengthened the effect of LXR-623 (Figure S2a). Furthermore, western blot analysis showed that LXR-623-induced upregulation in cyclin E1 and CDK2 expression and downregulation in cyclin A2 expression could be weakened through the knockdown of LINC01125 or magnified through the upregulation in LINC01125 expression (Fig. 4c).

### LXR-623 inhibits cell proliferation through the activation of the LINC01125/PTEN/AKT/MDM2/p53 axis

**Fig. 5:**
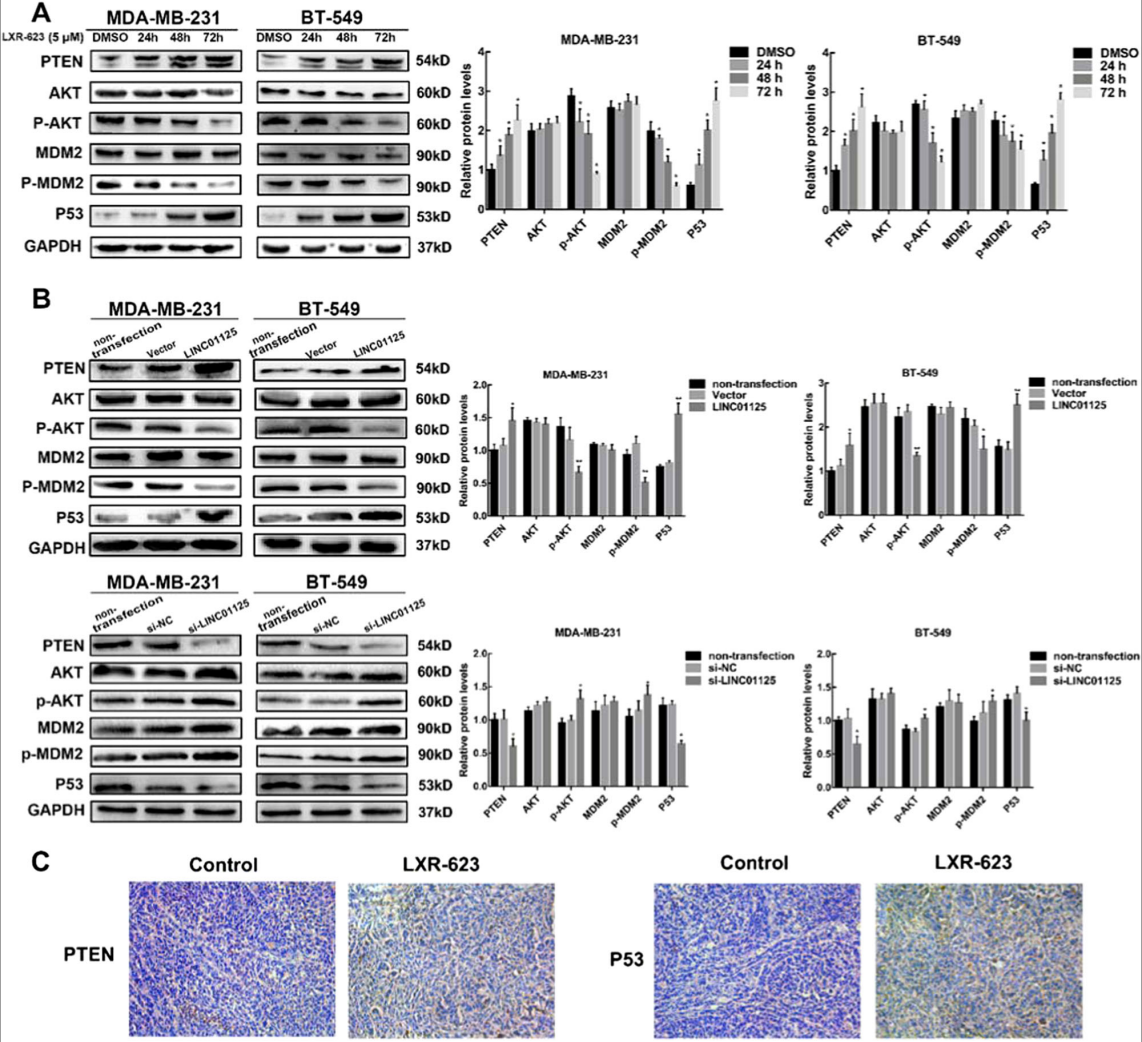
LXR-623 inhibits the proliferation via activating LINC01125/PTEN/AKT/MDM2/p53 axis. **a**, **b** Western blotting analysis was used to determine the effect of LXR-623 (5 μM) and LINC01125 on PTEN, AKT, p-AKT, MDM2, p-MDM2, and p53 expression levels in MDA-MB-231 and BT-549 cells. **c** The expression of PTEN and p53 in xenograft tumors by IHC assay. The data are shown as the mean ± SD of three replicates. *P < 0.05, **P < 0.01

To determine the mechanism underlying the anti-proliferation effect of LXR-623 in vitro, we performed western blot analysis. The results showed that the cells treated with LXR-623 at 5 μM concentration for 24, 48, and 72 h showed an increase in the expression of PTEN and p53 and reduced the expression of p-Akt and p-MDM2 in a time-dependent manner (Fig. 5a). Furthermore, the knockdown of LINC01125 reduced the expression of PTEN and p53 and increased the expression of p-Akt and p-MDM2, while LINC01125 overexpression enhanced the expression of PTEN and p53 and lowered the expression of p-Akt and p-MDM2 (Fig. 5b). Immunohistochemistry (IHC) assay showed that the expressions of PTEN and p53 were increased in the xenograft tumors (Fig. 5c).

### SF1670 partially prevents the anti-proliferation effect of LXR-623 and LINC01125

**Fig. 6:**
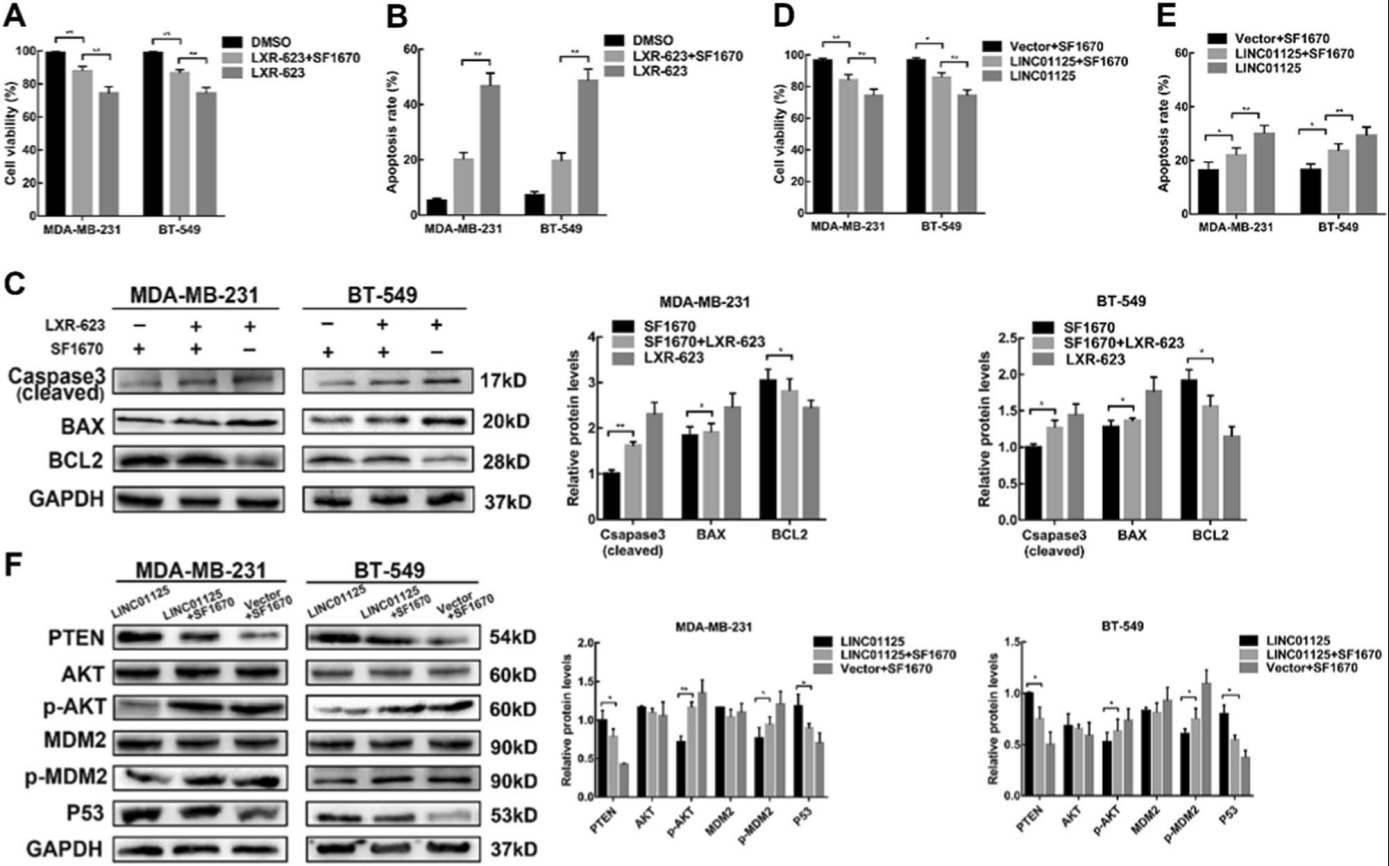
SF1670 partially prevents the anti-proliferation effect of LXR-623 and LINC01125. **a**, **b** The impact of SF1670 (3 μM) on cell proliferation when combined with or without LXR-623 (5 μM) by CCK8 assay and flow cytometry analysis. **c** Western blot analysis was used to determine the protein levels of cleaved Caspase3, BAX, and BCL2. **d**, **e** The effect of SF1670 (3 μM) on cell proliferation of cells transfected with or without LINC01125 overexpression vector by CCK8 assay and flow cytometry analysis. **f** Western blot detected the expression of PTEN/AKT pathways. The data are expressed as the mean ± SD of at least three independent experiments. *P < 0.05, **P < 0.01

To examine the regulation of PTEN pathways mediated by LXR-623, MDA-MB-231 and BT549 cells were treated with a PTEN specific inhibitor (SF1670) for 48 h. We tested the inhibitory effect of SF1670 at 1.5 and 3 μM concentrations for 48 h by western blotting and found that SF1670 could block the expression of PTEN and p53 and increase the levels of p-AKT and p-MDM2 (Figure S3a). Then, SF1670 treatment at 3 μM concentration could partly reverse the LXR-623-induced anti-proliferation effect, as revealed from the results of the CCK8 assay and apoptosis experiments (Fig. 6a, b). We found an increase in the expression of cleaved caspase-3 and BAX and a decrease in BCL-2 expression upon treatment with LXR-623. These effects of LXR-623 could be partly inhibited through SF1670 (Fig. 6c). The LINC01125-mediated anti-proliferation effect was also reversed by SF1670 (Fig. 6d, e). In addition, the BC cells treated with SF1670 showed an increase in the expressions of p-AKT and p-MDM2 and a decrease in the expressions of PTEN and p53 upon LINC01125 overexpression, suggestive of a link between LINC01125 and PTEN pathways (Fig. 6f).

### LINC01125 is a direct transcriptional target of p53

**Fig. 7:**
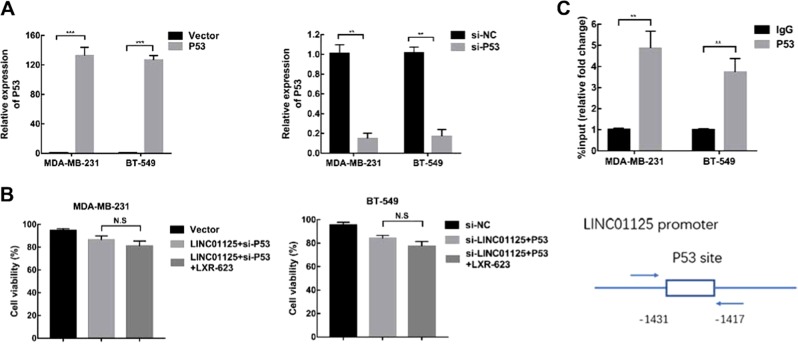
LINC01125 was a direct transcriptional target of p53. **a** The efficiency of overexpression and knockdown were verified by RT-qPCR in MDA-MB-231 and BT-549 cells were transfected with p53 vectors and siRNAs. **b** The combined effect of LXR-623 and co-transfection of pcDNA-LINC01125 + si-p53 or si-LINC01125 + pcDNA-p53 on cell viability was measured by CCK8 assay. **c** ChIP assays detected the enrichment of p53 in the LINC01125, and the promoter region was quantified by RT-qPCR. Data shown are mean ± SD of three independent experiments. **P < 0.01, ***P < 0.001, NS non-significant

To investigate the mechanism underlying the regulatory effect of LINC01125 on PTEN pathway, we examined the impact of p53 overexpression and knockdown on BC cell lines. Firstly, qRT-PCR results demonstrated that the expression of p53 was significantly induced or reduced in both MDA-MB-231 and BT549 cell lines after its overexpression or knockdown, respectively (Fig. 7a). CCK8 assay was used to evaluate the viability of the co-transfected MDA-MB-231 and BT549 cells. Treatment with LXR-623 had no effect on the co-transfection of LINC01125 overexpression vector and si-p53 as well as si-LINC01125 and p53 overexpression vector (Fig. 7b). Furthermore, ChIP assay was performed to evaluate the binding of p53 to the promoter region of LINC01125, with an IgG-precipitated sample used as a negative control. We observed an increase in the binding between p53 and the LINC01125 promoter in the two cell lines (Fig. 7c). These data suggest that p53 showed significant binding to the promoters of LINC01125.

## Discussion

Nuclear hormone receptor modulators could serve as potential cancer drugs. LXR modulators have gained attention owing to their actions against intestinal cancers, squamous cancer cells, BC cells, and prostate cancer cells^17–21^. Previous studies have shown that an LXR agonist, T0901317, inhibited the proliferation of ovarian cancer cells and tumor formation through the elevated expression of p27, a CDK inhibitor^22^. The metabolic targets of LXRs and their ligands, including GW3965 compound, may also be related to the anti-proliferation effects observed in several cancer cells. This effect may be mediated through the elevated levels of the cholesterol transporter ATP-binding cassette (subfamily A [ABC1], member 1 [ABCA1]) during carcinogenesis that inhibits low-density lipoprotein (LDL) uptake and induces cholesterol efflux^11,23^. Thus, targeting of LXRs with LXR agonists may serve as a strategy to suppress tumor growth.

LXR-623 is non-toxic and exerts anti-atherogenic activity on lipids. It was the first LXR agonist to enter clinical trials^24^. At present, little is known about the antitumor effect of LXR-623 and its underlying mechanism. To the best of our knowledge, this study is the first to show that LXR-623 may inhibit the proliferation of BC cells and regulate lncRNA expression. LXR-623 inhibits proliferation and induces apoptosis and cell cycle arrest in BC cells in a time- and concentration-dependent manner and suppresses tumor growth in xenograft mouse model, suggesting that LXR-623 may participate in the regulation of apoptosis-related pathways.

Evidences have indicated that lncRNAs are essential regulators involved in the control of the fundamental biological processes^25–27^. Here, we investigated lncRNA expression profiles in response to LXR-623 treatment in MDA-MB-231 cells and identified LXR-623-induced upregulated expression of the lncRNA, LINC01125. The analysis of TCGA database and tissues samples of patients with BC demonstrated the significant downregulation in LINC01125 expression. The gain- and loss-of-function experiments showed that LINC01125 not only displays the characteristics of tumor suppressor gene but also blocks the inhibitory effects of LXR-623 on cell proliferation through the reduction in LINC01125 expression.

AKT signaling is a fundamental pathway that regulates multiple biological processes, including apoptosis and cell growth, and is known to be hyperactivated in various cancers^28^. Kyoto Encyclopedia of Genes and Genomes (KEGG) pathway analysis (Supplementary Figure S1B) demonstrated that the AKT pathway is associated with LXR-623-regulated differentially expressed lncRNAs. In addition, PTEN, a crucial tumor suppressor, is a prime antagonist of phosphatidylinositol-4,5-bisphosphate 3-kinase (PI3K) and promotes AKT degradation. PTEN acts as a negative regulator of this pathway^29–33^. After treatment with SF1670, a PTEN inhibitor, the anti-proliferative effect of LXR-623 was greatly suppressed. As LXR-623 and LINC01125 depend on the PTEN/AKT/MDM2/p53 axis to suppress the proliferation of cancer cells, it is important to understand the mechanism underlying the effect of LINC01125 on the PTEN/AKT/MDM2/p53 pathway. We carried out a promoter prediction analysis of possible transcription factors that could regulate LINC01125 expression and found that the transcription factor, p53, was a good candidate. ChIP assay results also illustrated that LINC01125 was a direct transcriptional target of p53 in BC cell lines. Collectively, these results demonstrate that LINC01125 may serve as a coactivator of p53 to stimulate the PTEN pathway expression for mediating the LXR-623-induced anti-proliferative effect.

Additional studies are warranted to evaluate the relationship between LXR-related lncRNAs and lipid metabolism in antitumor activities. The lncRNA LeXis was recently shown to promote cholesterol efflux and simultaneously block the cholesterol biosynthesis through the activation of LXRs^34^. Moreover, the metabolism of glucose, glutamine, and lipid is essential for tumor progression, and the tumor suppressor p53 is a transcription factor involved in cellular metabolism and appears to play a key role in tumor suppressive activities^35^. Whether LXR-623-induced lncRNA regulates the expression of p53 transcription factors to modulate lipid metabolism may be investigated in future studies.

In our study, the LXR-623-p53-LINC01125-PTEN/AKT axis showed promising antitumor effects on BC cells and provided a new insight into the chemopreventive mechanisms associated with LXR agonists. Further delineation of the molecular mechanisms underlying the effects of PTEN and AKT/p53 signaling on the differential modulation of tumor cell metabolism may improve the understanding of the link between genetic alterations and cellular metabolism in cancer and contribute to more effective and less toxic treatments.

## Materials and methods

### Cell culture, siRNA, plasmid construction, and transfection

The human cell lines MCF-10A, MDA-MB-231, and BT549 were obtained from the American Type Culture Collection (ATCC; Manassas, VA, USA), while MCF-7 and MDA-MB-453 cell lines were purchased from the Type Culture Collection of the Chinese Academy of Sciences (Shanghai, China). MDA-MB-231, MDA-MB-453, and MCF-7 cells were cultured in Dulbecco’s modified Eagle’s medium (DMEM; Gibco, Carlsbad, CA, USA) and BT549 cells were cultivated in Roswell Park Memorial Institute (RPMI)-1640 (Gibco) medium supplemented with 10% fetal bovine serum (FBS, Gibco). MCF-10A cells were cultured in MEBM BulletKit (Lonza, Basel, Switzerland). All cells were incubated in a humified 5% CO_2_ incubator at 37 °C. Commercialized si-LINC01125, si-p53, and negative-control siRNA as well as pCDNA3.1-LINC01125 and pCDNA3.1-p53 plasmid were purchased from Thermo Fisher Scientific (MA, USA). MDA-MB-231 and BT549 cells were seeded in six-well plates at a density of 3 × 10^5^ cells/well overnight. Cell transfection was performed with Lipofectamine 2000 (Invitrogen, Carlsbad, CA, USA) according to the manufacturer’s protocol. The sequences of siRNAs are presented in Supplementary Table S1. To knock down LINC01125, three siRNAs and a siRNA-NC were examined by qRT-PCR and siRNA-3 as the most effective one (Supplementary Figure S1A).

### Cell viability assay

Cell viability was assessed with the CCK8 colorimetric assay (Bosterbio, Wuhan, China). Briefly, cells were initially transfected with an siRNA or indicated vector or co-transfected with siRNA and vector. At 48 h after transfection, cells (2 × 10^3^ cells/well) were cultured overnight in 96-well plates. The medium was replaced with that supplemented with LXR-623 or DMSO at indicated concentrations. After incubation for the indicated time, 10 µL of CCK8 reagent was added to each well and the cells were cultured for 2 h at 37 °C. The absorbance was recorded at 490 nm wavelength using a microplate reader (Multiskan™ GO microplate spectrophotometer; Thermo Fisher Scientific, Inc.). Cell viability was calculated according to the following formula: Cell viability (%) = (OD treatment − OD blank)/(OD control − OD blank).

### Apoptosis assay

Annexin V-FITC/propidium iodide (PI) Apoptosis Detection Kit (Beyotime, Haimen, China) was used to study the mechanism of cell death according to the manufacturer’s instructions after drug treatment or cell transfection. Cells (5 × 10^5^ cells/mL) were resuspended and mixed in 500 μL of a binding buffer with 5 μL of Annexin V-FITC and 5 μL of PI. After incubation for 15 min, cell apoptosis was tested by flow cytometry (NovoCyte, ACEA Biosciences, San Diego, CA, USA) and data analyses were performed by FlowJo.

### Cell cycle analysis

To study the effect of LXR-623 and transfection with siRNA or indicated vector on cell cycle progression, cells were trypsinized and washed with phosphate-buffered saline (PBS), followed by treatment with 70% cold absolute ethanol (added drop-wise). The cells were incubated at −20 °C for at least for 24 h and then stained with 50 µg/mL of PI (Sigma-Aldrich, St. Louis, USA) in the presence of 100 µg/mL RNAse (Sigma) at 37 °C for 30 min. The stained nuclei were analyzed on BD-FACSAria flow cytometer (BD Biosciences, CA, USA).

### Colony formation assay

Cells were cultured in six-well plates (0.5 × 10^3^ cells/well) in a medium supplemented with LXR-623 at various concentrations (1, 2.5, and 5 μM) or DMSO for 14 days, followed by treatment with 10% formaldehyde for 5 min. Cells were stained with 1% crystal violet for 1 min before calculating the number of colonies.

### Xenograft tumor model

All animal experiments were approved by the Chongqing Medical University Animal Care and Use Committee and performed in accordance with the guidelines of the National Institutes of Health. Female BALB/c mice (4–6-week-old, 18–20 g) were subcutaneously injected with MDA-MB-231 cells (2 × 10^6^) in 100 µL of sterile PBS in the dorsal region. Animals were randomly divided into two groups and treatment was started on the 2nd day. Once the tumors reached a measurable size of approximately 100 mm^3^ in all mice, mice were either treated with the vehicle (0.5% methylcellulose, 2% Tween-80 in water) or LXR-623 (50 mg/kg) suspended in vehicle via oral gavage daily for 10 days. Tumor volume and body weight were evaluated every other day during the treatment period. Tumor volumes were measured with electronic digital calipers and determined by measuring length (*A*) and width (*B*) to calculate volume (*V* = *AB*^2^/2).

### Microarray of lncRNA

Total RNA was extracted from MDA-MB-231 cells treated with DMSO or LXR-623 (5 μM) for 48 h. Expression profiling of lncRNAs was performed using the Agilent human lncRNA array V.2.0 platform (Agilent Technologies, Santa Clara, CA, USA). The results of the microarray analysis were used for investigating the candidate target genes.

### Tissue sample

The 15 pairs of BC tissues and adjacent non-cancerous tissues included in this study were obtained from the patients at the First Affiliated Hospital of Chongqing Medical University (Chongqing, China). None of the patients received chemotherapy or radiotherapy treatment before surgery. Tissues were collected after surgical resection and immediately frozen in liquid nitrogen and stored until further use. The present study was approved by the Ethics Committee of Chongqing Medical University and performed in accordance with the Declaration of Helsinki.

### RNA extraction, qRT-PCR, and western blot assays

Total RNA was extracted from cells using TRIzol reagent (Invitrogen) and cDNAs were synthesized using the PrimeScript reverse transcriptase reagent kit (TaKaRa, Shiga, Japan) following the manufacturer’s instructions. Quantitative real-time PCR was carried out with SYBR Green (TaKaRa) and the data were assessed on the CFX Connect Real-time System (BIO-RAD, CA, USA). Gene expression was evaluated with the 2^−ΔΔCT^ method and the CT value was compared with that for glyceraldehyde-3-phosphate (GAPDH). All experiments were repeated at least thrice. The primer sequences are shown in Supplementary Table 1.

Cells were washed twice with PBS and lysed in 100 μL of radioimmunoprecipitation assay (RIPA) buffer (Bosterbio) containing protease inhibitors. Cell protein lysates were separated by 10% sodium dodecyl sulfate polyacrylamide gel electrophoresis (SDS-PAGE) and transferred onto 0.22-μm polyvinylidene fluoride (PVDF) membranes (Sigma). The membranes were blocked with bovine serum albumin (BSA) for 2 h at room temperature and probed with rabbit primary antibodies (Supplementary Table 2). Then washed the membrane and followed by incubation with secondary antibodies (Cell Signaling Technology, Beverly, MA, USA) at room temperature for 1 h. Densitometry (Quantity One software; Bio-Rad) was used to quantify the autoradiograms.

### Immunohistochemistry (IHC)

For IHC, paraffin-embedded slides were deparaffinized, rehydrated, and stained with antibodies against PTEN (1:150) and p53 (1:150) at 4 °C overnight. The slides were treated with a secondary antibody, followed by incubation with 3,3′-diaminobenzidine (DAB). The slides were counter stained with hematoxylin and observed under a light microscope.

### RNA fluorescent in situ hybridization (FISH)

RNA FISH assay was performed with a Fluorescent in Situ Hybridization Kit (RiboBio, China) following the manufacturer’s suggestions. In order to achieve a sufficient signal-to-background ratio, multiple probes were targeted to each lncRNA sequence. A set of 15–20 probes that covered the entire length of the RNA molecule provided the optimal signal strength, and each probe carried multiple fluorophores. The pooled FISH probes were resuspended at a final concentration of 25 μM in an RNase-free storage buffer and protected from light at −20 °C. The FISH results were captured using a confocal instrument (Leica TCS-SP8).

### Chromatin immunoprecipitation (ChIP)

The ChIP assay was performed using the ChIP assay kit according to the manufacturer’s protocol (Cell Signaling Technology). Cells were crosslinked with 1% (v/v) formaldehyde for 10 min at room temperature and the reaction was terminated with 125 mM glycine treatment for 10 min. The extracted chromatin was digested, sonicated, and fragmented into 150–900 bp. Chromatin extracts were immunoprecipitated with anti-p53 and anti-IgG antibodies on Protein-A/G-Sepharose beads. After washing, elution, and de-crosslinking, PCR was performed using the primers spanning the putative p53-binding site on LINC01125 promoter.

### Chemicals

LXR-623 and SF1670 were purchased from MedchemExpress (Monmouth Junction, NJ, USA).

### Statistical analysis

All experimental assays were independently repeated in triplicates and data were expressed as mean ± standard deviation (SD). Two-tailed Student’s *t*-test and one-way analysis of variance (ANOVA) were used to assess the statistical difference between groups. All statistical data were evaluated using Statistical Program for Social Sciences 19.0 software (SPSS, Palo Alto, CA, USA) and presented with GraphPad Prism 7.0 (GraphPad Software, La Jolla, CA, USA). A value of *P* less than 0.05 was considered as significant.

## Supplementary information

Supplementary Table 1 and Table 2

Figure S1, Figure S2, Figure S3, Figure S4A and S4B, and Figure S5
