# Supplementary material for: The LXR-623-induced long non-coding RNA LINC01125 suppresses the proliferation of breast cancer cells via PTEN/AKT/p53 signaling pathway
Source: Cell Death Dis. 2019 Mar 13;10(3):248. doi: 10.1038/s41419-019-1440-5 (PMC6416354; doi:10.1038/s41419-019-1440-5)
Supplement: Supplementary file 1 — Supplementary Table 1 and Table 2 [file 41419_2019_1440_MOESM1_ESM.docx]

**Supplementary Tables**

**Supplementary Table 1. Primer sequences and target sequences used in this study**

| **Gene** | **Sequence or Target Sequence** |
| --- | --- |
| LINC01125-F | 5'-TTCTCCATCTGCGCACCACA-3' |
| LINC01125-R | 5'-GCCAGCCATCGGTGCCATAT-3' |
| GAPDH-F | 5'-CACCCACTCCTCCACCTTTG-3' |
| GAPDH-R | 5'-CCACCACCCTGTTGCTGTAG-3' |
| LINC01125-siRNA #1 | 5'-GCAGUCAGAUCCAUUACAATT-3' |
| LINC01125-siRNA #1 | 5'-UUGUAAUGGAUCUGACUGCTT-3' |
| LINC01125-siRNA #2 | 5'-CCAUCAUUUAACGAAGCAATT-3' |
| LINC01125-siRNA #2 | 5'-UUGCUUCGUUAAAUGAUGGTT-3' |
| LINC01125-siRNA #3 | 5'-GGGAUUUAAGAGAGCAGUUTT-3' |
| LINC01125-siRNA #3 | 5'-AACUGCUCUCUUAAAUCCCTT-3' |
| LINC01125siRNA-NC-F | 5'-UUCUCCGAACGUGUCACGUTT-3' |
| LINC01125siRNA-NC-R | 5'-ACGUGACACGUUCGGAGAATT-3' |
| p53-F | 5'-GAGGATTCACAGTCGGATA-3' |
| p53-R | 5'-ATCATCTGGAGGAAGAAGTT-3' |
| p53-siRNA | 5'-CUACUUCCUGAAAACAACGTT-3' |
| p53-siRNA | 5'-CGUUGUUUUCAGGAAGUAGTT-3' |
| p53-siRNA-NC-F | 5'-UUCUCCGAACGUGUCACGUTT-3' |
| p53-siRNA-NC-R | 5'-ACGUGACACGUUCGGAGAATT-3' |
| p53 binding sequence | 5'-TGAAATGCAACAAAAGGTACAC-3' |
|  | 5'-ATAACCACCACCACCAAAAAAT-3' |
|  |  |

**Supplementary Table 2. Antibodies used in this study**

| **Antibody (Item No.)** | **Specificity** | | |  | **Company** | | |
| --- | --- | --- | --- | --- | --- | --- | --- |
|  | **WB** | **IHC** | **ChIP** | |  |  |  |
| GAPDH(D16H11) | 1:1000 |  |  | | Rabbit monoclonal | Cell Signaling Technology |  |
| Cyclin E1(D7T3U) | 1:500 |  |  | | Rabbit monoclonal | Cell Signaling Technology |  |
| Cyclin A2(GTX103042) | 1:500 |  |  | | Rabbit monoclonal | GeneTex |  |
| CDK2(GTX133862) | 1:500 |  |  | | Rabbit monoclonal | GeneTex |  |
| BCL2(D55G8) | 1:500 |  |  | | Rabbit monoclonal | Cell Signaling Technology |  |
| cleaved Caspase-3(D175) | 1:1000 |  |  | | Rabbit monoclonal | Cell Signaling Technology |  |
| BAX(D2E11) | 1:500 |  |  | | Rabbit polyclonal | Cell Signaling Technology |  |
| PTEN(138G6) | 1:500 | 1:150 |  | | Rabbit monoclonal | Cell Signaling Technology |  |
| AKT(C67E7) | 1:1000 |  |  | | Rabbit monoclonal | Cell Signaling Technology |  |
| p-AKT(S473) | 1:1000 |  |  | | Rabbit monoclonal | Cell Signaling Technology |  |
| MDM2(E1A6376) | 1:1000 |  |  | | Rabbit monoclonal | EnoGene Biotechnology |  |
| p-MDM2(E1A3376) | 1:1000 |  |  | | Rabbit monoclonal | EnoGene Biotechnology |  |
| P53(7F5) | 1:1000 | 1:150 | 1:200 | | Rabbit monoclonal | Cell Signaling Technology |  |
