# Supplementary material for: The LXR-623-induced long non-coding RNA LINC01125 suppresses the proliferation of breast cancer cells via PTEN/AKT/p53 signaling pathway
Source: Cell Death Dis. 2019 Mar 13;10(3):248. doi: 10.1038/s41419-019-1440-5 (PMC6416354; doi:10.1038/s41419-019-1440-5)

**Supplementary figures**

**Figure S1 LXR-623 suppresses the proliferation in breast cell lines. a** Flow cytometry analysis showed LXR-623 (5 μM) induces cell cycle arrest. The data were shown as mean ± SD for 3 separate experiments. **P*< 0.05, ***P*< 0.01.

**
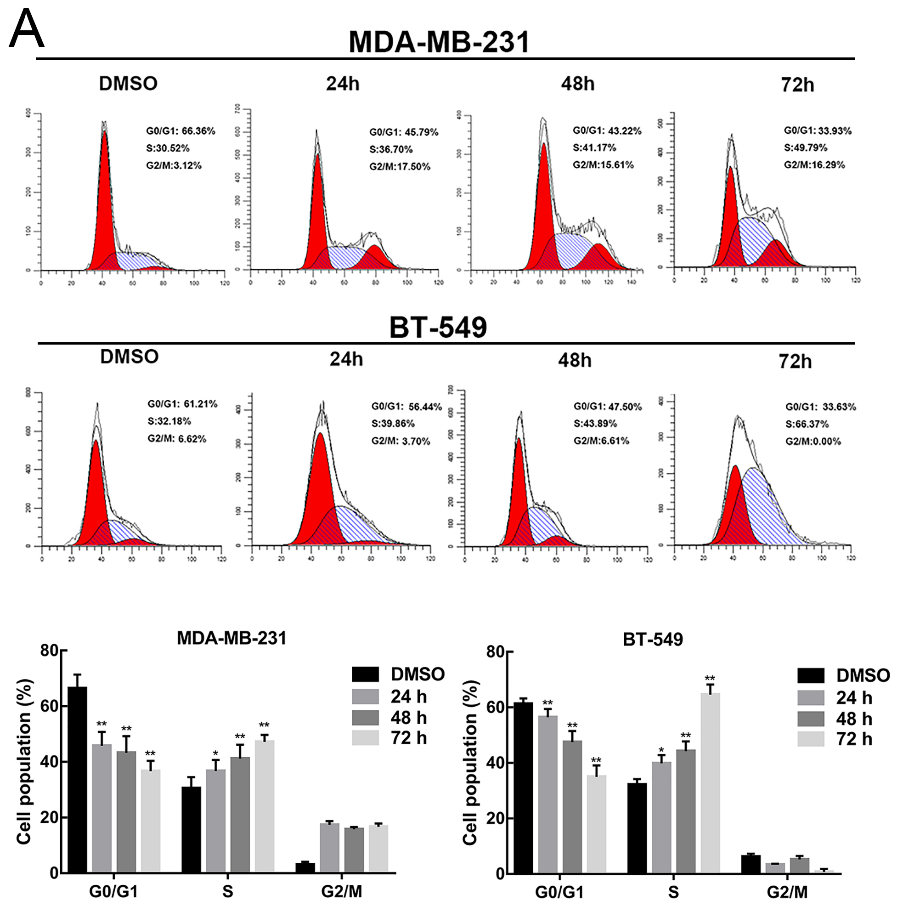
**

**Figure S2 LINC01125 plays a critical role in the LXR-623-induced apoptosis. a** Effect of LINC01125 and combined effect of LXR-623 (5 μM) and LINC01125 siRNAs or overexpression vectors on cell cycle arrest measured by Flow cytometry analysis. The data were shown as mean ± SD for 3 separate experiments, **P*< 0.05.

**
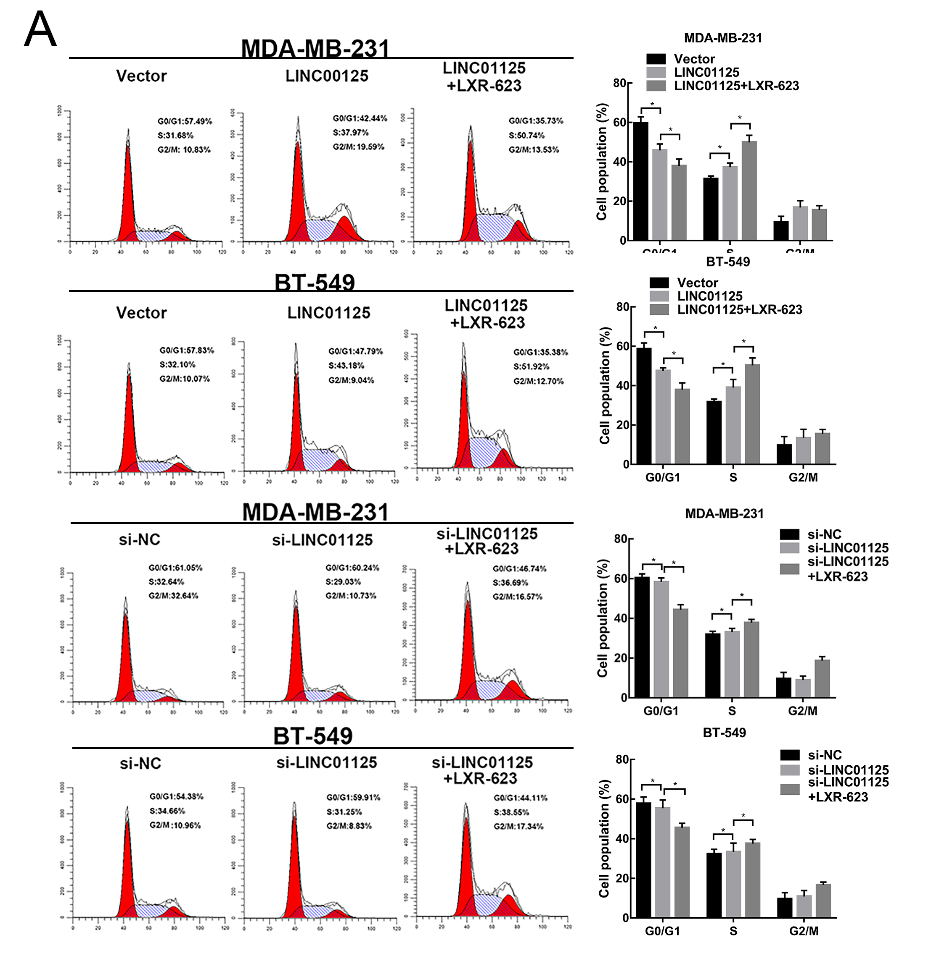
**

**Figure S3 Validation of the inhibitory effectiveness of SF1670. a** Western blot results verified that the SF1670 inhibitory effect at 1.5, 3 μM on PTEN/AKT/p53 pathways in MDA-MB-231 and BT-549 cells. The data are shown as the mean ± SD of three replicates; *P<0.05, **P<0.01


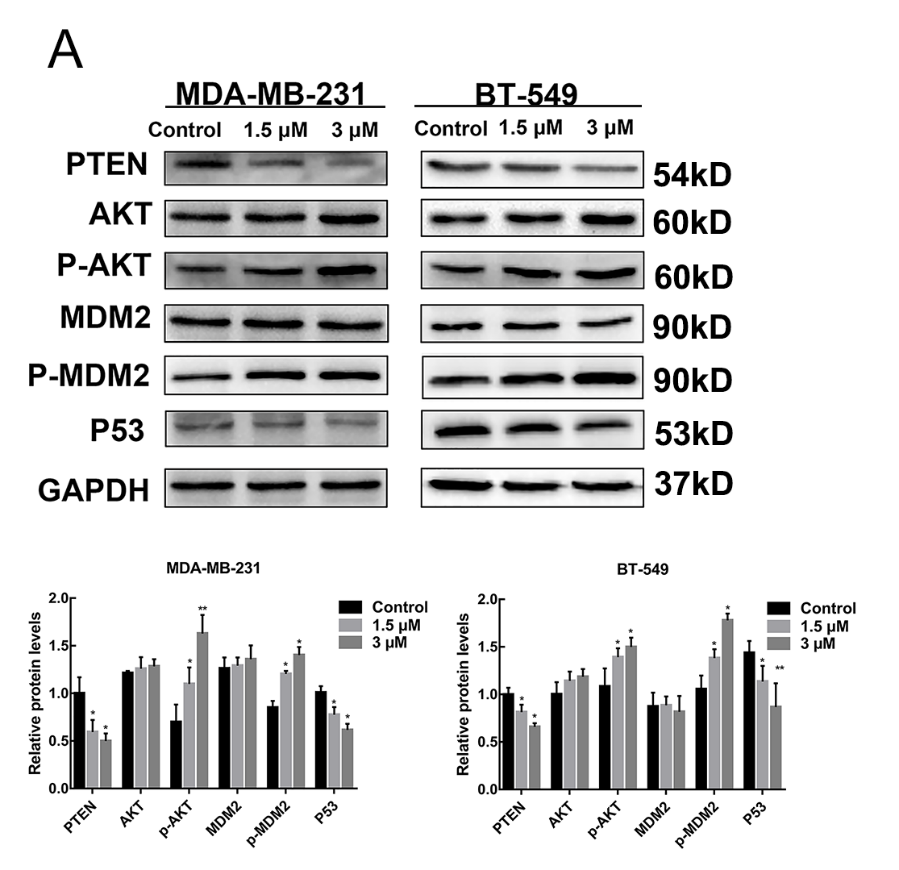


**Figure S4 Validation of the effectiveness of LINC01125. a** qRT-PCR was used to examined the LINC01125 relative expression in MDA-MB-231 and BT-549 cells transfected with indicated siRNAs. **b** MDA-MB-231 and BT-549 cells were transfected with LINC01125 vectors and siRNAs, and the efficiency of overexpression and knockdown were verified by RT-qPCR. *P<0.05, **P<0.01, ***P<0.001


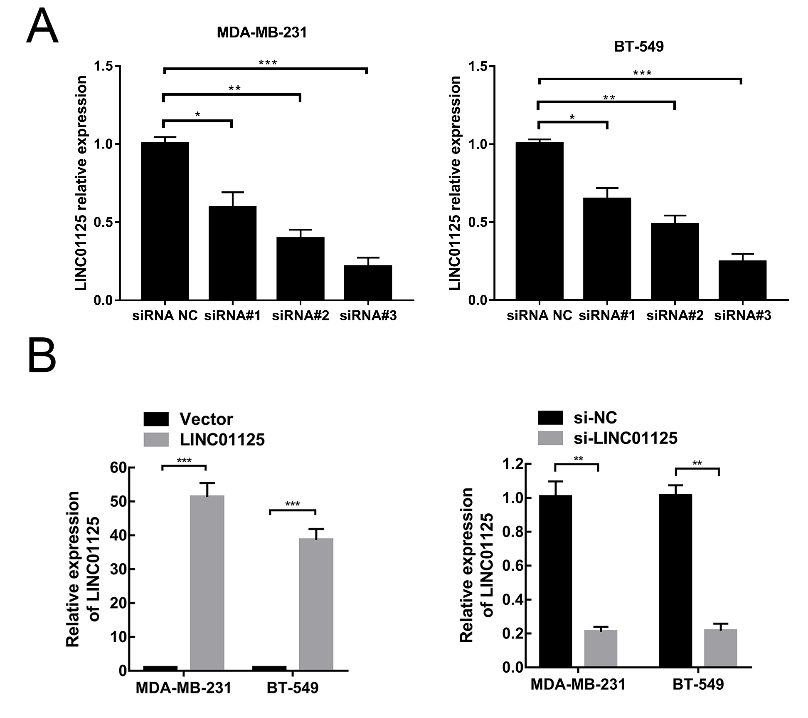


**Figure S5 KEGG analysis.** KEGG analysis of LXR-623-regulated differentially expressed lncRNAs.


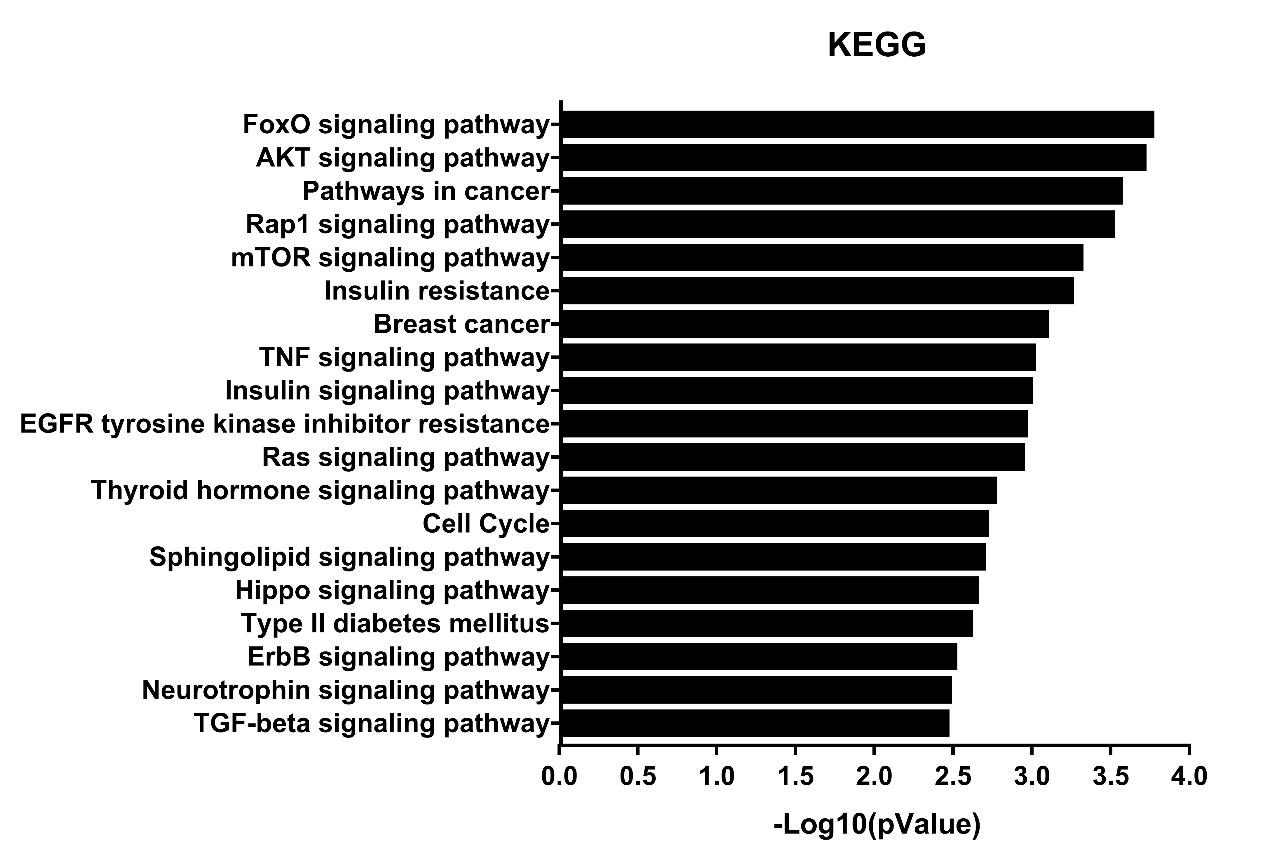

Supplement: Supplementary file 2 — Figure S1, Figure S2, Figure S3, Figure S4A and S4B, and Figure S5 [file 41419_2019_1440_MOESM2_ESM.docx]
